# The Great American Blight

**Published:** 1905-10

**Authors:** 


### TEXAS MEDICAL JOURNAL.

AUSTIN, TEXAS.

A MONTHLY JOURNAL OF MEDICINE AND SURGERY.

EDITED AND PUBLISHED BY
F. E. DANIEL, M. D.
Mrs. F. E. DANIEL, Business Manager.

Published Monthly at Austin, Texas. Subscription price $1.00 a year in advance

### THE GREAT AMERICAN BLIGHT.

APPEAL TO THE PRESIDENT.

The indiscriminate and almost universal use of patent medicines by all classes, of every age, and of both sexes—self-dosing—is a blight upon the American people, ruinous to body, mind and morals; ruinous not only in its immediate effects, but far-reaching and ruinous to coming generations. It is the crying evil of the day and calls more loudly than any other evil for reform.

This incubus, more deadly than the lethean shade of the Upas tree, has fastened its hold upon the people with grips of steel, and it seems that nothing can shake it off. The rich proprietors of alcoholic and opium and cocaine nostrums and abortion pills laugh in their sleeve at the puny efforts of the medical profession to regulate the sale, and I may say, of revenue agents, to correct the evil. There is so much money behind it that legislation against it, either State or National, is rendered impossible. These people know where to put the money, and there is no doubt that it is put where it will effectually stop proceedings.*

The all-powerful allies of the poisonous nostrums are the newspapers, largely the church papers. They exploit these poisons to the ignorant, credulous and frightened people, largely women. Some of them sell their space and even their news columns and editorial pages, to assist in foisting upon the people these death

*Bills to restrict this nefarious traffic were introduced simultaneously in thirteen State Legislatures last winter, and defeated in every one.

dealing frauds, and the papers and the proprietors are backed by the wholesale druggists and the retail druggists whose stock is two-thirds patent medicines. And yet, the majority of doctors continue to patronize them, send them their prescriptions, thus helping to maintain and spread the use of these infamous preparations. What will not some men do for money? I know doctors who accept a percentage on their prescriptions and who occupy furnished and equipped offices, the rent of which is paid by the prescription druggist, in consideration of prescription patronage. Some of this kind of doctors are loudest in the condemnation of the use of legitimate pharmacal preparations, yet they all use them.

In support of the above allegations I cite the papers that published that "interview with Dr. Hartman," in which, while the "mosquito theory" was endorsed and the methods of dealing with yellow fever were approved the "doctor" urged the use, constantly, of Peruna, as a preventive and cure for yellow fever. To thus work upon the fears of a stricken people to force them to buy a stuff that is said to be 47 per cent of alcohol, is infamous and should be a crime under our laws. The Journal of the A. M. A. recently gave editorially an account of a prominent gentleman, a total abstainer, a model of propriety, who was found by his physician who had been called, to be drunk, and moreover suffering from chronic alcoholism, from the free use of Peruna. In Austin, a girl suffering from dysmenorrhea, was in the habit of taking Peruna, and being in a maudling state and hysterical, her family sent for the doctor (Dr. G. H. W.). They were shocked when the doctor told them the girl was drunk.

Can such things be? Will an enlightened government longer tolerate this slow process of poisoning our people—the fathers and mothers of coming generations, the poisoning of the social stream at its fountain and source? Ah, but how can it be stopped? Congress has persistently refused to create a Public Health Commission or a National Board of Health, the only tribunal capable of dealing with matters that affect the public health and morals; every effort to regulate this traffic by legislation or congressional enactment has been defeated, by means that are well understood, though no man dare to openly charge bribery, however much it may be suspected; the revenue agents who would proscribe the alcoholic nostrums and put them on the liquor list are not sustained by the State governments (I will give an instance presently), and the Postoffice Department in face of the showing of fraud in certain advertisements will not act. Mr. Bock (*Ladies' Home Journal,* September, 1905), shows a "congressman's testimonial to Peruna" side by side with the congressman's letter stating that it is a forgery and that neither he nor his family ever tasted the stuff. Mr. Bock also gives a picture of Mrs. Lydia E. Pinkham's tombstone showing that she died twenty-two years ago, side by side with the extract from the Pinkham advertisement where Mrs. P. is still calling on her afflicted sisters to buy the vegetable compound and be healed. If these things are not frauds, what are they? Should the United States mails be made a party to the sale of stuff that poisons and ruins our people? The drink habit and the morphine habit and the cocaine habit and the abortion habit, the result of the use of these nostrums, will tell upon the next and succeeding generations; they will be neurasthenics, consumptives, imbeciles. Doubtless the Great American Blight is a factor in the alarming increase of insanity in Texas to which Dr. M. L. Graves, late superintendent of the Southwest Texas Insane Asylum, calls attention in Texas Medical Journal for April 1905, an increase at a ratio of 13.75 to 1 of population in the last forty-five years.

The instance of defeat of an honest and well-meaning revenue officer referred to above, occurred in Texas about three years ago. When Judge Cunningham was State revenue officer of Texas, he promulgated an order that Peruna and other fancy drinks sold under a most diaphanous guise of "medicine" should pay liquor dealer's license. But *somebody* had influence enough with *somebody* having the authority, to get the ruling set aside; I do not know how, or by whom it was done; I state but the facts as given me by Judge Cunningham (He resigned [?]—then), and by General Flanagan, the United States Internal Revenue Collector for Texas. And now, the latest effort, a very feeble and ineffectual one it will be, to put these fancy drinks on the liquor dealers' license and tax them as liquor, has been made by Mr. Yerkes, the United States Commissioner of Internal Revenue. Read it in this issue. It will be seen that there is a gap in it large enough to permit a Texas steer to jump through, a rent in the drag net through which every one of the fancy drinks can escape. The proprietor has only to show that his cocktail of mean whisky "contains medicinal substances," if only a little willow bark.

Well, what are we going to do about it? Let us bring it directly to the attention of our great, wise, courageous and honest President, and ask in the name of humanity for relief and deliverance from the Great American Blight. It is *race suicide!* Let the American Medical Association, the Public Health Association, the W. C. T. U., all the Y. M. C. A. Associations, all the Alumni Associations of our great Universities, join in a personal memorial to the President. He, at least, can not be corrupted nor hood-winked. Who will start the movement? The people have a right to appeal to the President when they can not get relief otherwise. Let's invoke the big stick, for the protection of ignorant, credulous, frightened and outraged people who are duped or driven or frightened into the use of death-dealing nostrums.

